# Mucin expression in gastric- and gastro-oesophageal signet-ring cell cancer: results from a comprehensive literature review and a large cohort study of Caucasian and Asian gastric cancer

**DOI:** 10.1007/s10120-020-01086-0

**Published:** 2020-06-02

**Authors:** K. G. P. Kerckhoffs, D. H. W. Liu, L. Saragoni, R. S. van der Post, R. Langer, M. Bencivenga, M. Iglesias, G. Gallo, L. C. Hewitt, G. E. Fazzi, A. M. Vos, F. Renaud, T. Yoshikawa, T. Oshima, A. Tomezzoli, G. de Manzoni, T. Arai, R. Kushima, F. Carneiro, H. I. Grabsch

**Affiliations:** 1 Department of Pathology, GROW School for Oncology and Developmental Biology, Maastricht University Medical Center+, P. Debyelaan 25, 6229HX Maastricht, The Netherlands; 2 Pathology Unit, Morgagni-Pierantoni Hospital, Forlì, Italy; 3 Department of Pathology, Radboudumc, Nijmegen, The Netherlands; 4 Institute of Pathology, University of Bern, Bern, Switzerland; 5 Unit of General and Upper GI Surgery , University of Verona, Verona, Italy; 6 Pathology Department, Hospital del Mar, Universitat Autonoma de Barcelona, Barcelona, Spain; 7 Department of Anatomic Pathology, Azienda Ospedaliero-Universitaria Policlinico di Modena, Modena, Italy; 8 Department of Pathology, Univ. Lille, CNRS, Inserm, CHU Lille, UMR9020 – UMR-S 1277 - Canther – Cancer Heterogeneity, Plasticity and Resistance to Therapies, Lille, France; 9 Department of Gastric Surgery, National Cancer Center Hospital, Tokyo, Japan; 10 Department of Gastrointestinal Surgery, Kanagawa Cancer Center, Yokohama, Japan; 11 Department of Pathology, Verona University Hospital, Verona, Italy; 12 Department of Pathology, Tokyo Metropolitan Geriatric Hospital and Institute of Gerontology, Tokyo, Japan; 13 Department of Pathology, Shiga University of Medical Science, Shiga, Japan; 14 Institute of Molecular Pathology and Immunology at the University of Porto (Ipatimup), Porto, Portugal; 15 Instituto de Investigação e Inovação em Saúde (i3S), University of Porto, Porto, Portugal; 16 Pathology Department, Centro Hospitalar de São João and Faculty of Medicine, Porto, Portugal; 17 Division of Pathology and Data Analytics, Leeds Institute of Medical Research at St. James’s, University of Leeds, Leeds, UK

**Keywords:** Gastric cancer, Signet-ring cells, Mucin, Histological phenotype, Survival

## Abstract

**Background:**

The literature on the prognostic relevance of signet-ring cell (SRC) histology in gastric cancer (GC) is controversial which is most likely related to inconsistent SRC classification based on haematoxylin–eosin staining. We hypothesised that mucin stains can consistently identify SRC-GC and predict GC patient outcome.

**Methods:**

We performed a comprehensive literature review on mucin stains in SRC-GC and characterised the mucin expression in 851 Caucasian GC and 410 Asian GC using Alcian Blue (AB)-Periodic Acid-Schiff (PAS), MUC2 (intestinal-type mucin), and MUC5AC (gastric-type mucin). The relationship between mucin expression and histological phenotype [poorly cohesive (PC) including proportion of SRCs, non-poorly cohesive (non-PC), or mucinous (MC)], clinicopathological variables, and patient outcome was analysed.

**Results:**

Depending on mucin expression and cut-offs, the positivity rates of SRC-GC reported in the literature varied from 6 to 100%. Patients with MUC2 positive SRC-GC or SRC-GC with (gastro)intestinal phenotype had poorest outcome.

In our cohort study, PC with ≥ 10% SRCs expressed more frequently MUC2, MUC5AC, and ABPAS (*p* < 0.001, *p* = 0.004 and *p* < 0.001, respectively). Caucasians with AB positive GC or combined ABPAS-MUC2 positive and MUC5AC negative had poorest outcome (all *p* = 0.002). This association was not seen in Asian patients.

**Conclusions:**

This is the first study to suggest that mucin stains do not help to differentiate between SRC-GC and non-SRC-GC. However, mucin stains appear to be able to identify GC patients with different outcome. To our surprise, the relationship between outcome and mucin expression seems to differ between Caucasian and Asian GC patients which warrants further investigations.

**Electronic supplementary material:**

The online version of this article (10.1007/s10120-020-01086-0) contains supplementary material, which is available to authorized users.

## Introduction

Gastric cancer (GC) is a heterogenous disease with respect to epidemiology, morphology, and clinical behaviour. Despite declining incidence, GC remains one of the major causes of cancer-related death worldwide [1]. The incidence of poorly cohesive GC, including signet-ring cell (SRC) cancer, appears to be rising in the Western World [2, 3]. Several studies investigating the prognostic relevance of SRC histology reported conflicting results [4–8]. In some studies, SRC histology was associated with poor outcome, which was not confirmed in other studies [4–10]. Furthermore, it has been suggested that the relationship between SRC histology and outcome may depend on the disease stage in GC patients [11–17]. The clinical utility of the proportion of SRCs to predict response to preoperative chemo(radio)therapy in GC patients remains a matter of debate [18–24].

A recent expert panel hypothesised that these inconclusive results could be related to inconsistencies in the histological classification of SRC-GC [25]. Whilst SRC-GC have always been typed as diffuse-type cancers in the Lauren classification [26], the WHO definition of SRC-GC changed several times between the 1st edition in 1977 [27] and the 4th edition in 2010 [28]. Up to the 4th edition [27, 29, 30], when SRC-GC became a subcategory of poorly cohesive GC, SRC-GC was classified as a separate specific subtype of GC. Furthermore, the definition of the extent of SRCs to qualify as SRC-GC changed over the years from ‘predominant’, to more than 50% SRCs in the 2nd edition WHO [29] and back to “predominantly” or “exclusively” in the 4th and 5th editions WHO [28, 31]. In an attempt to achieve a more consistent classification of SRC-GC, a multidisciplinary expert panel recently proposed specific cut-off values for the percentage of SRCs to distinguish bona-fide SRC-GC (more than 90% SRCs) from poorly cohesive GC with SRC component (between 10 and 90% SRCs) and poorly cohesive GC not otherwise specified (less than 10% SRCs) [25]. However, what remains particularly challenging is the unequivocal definition of what constitutes a SRC based on routine histology as exemplified by the 5 different types of SRCs described in the 3rd edition WHO [30]. Therefore, there remains an urgent clinical need to identify a specific biomarker for SRCs to standardise SRC-GC classification and establish the clinical importance of SRC-GC.

We hypothesised that (1) SRC containing gastric cancers have a different mucin expression compared to non-SRC gastric cancers, and (2) there is an association between SRC mucin expression, clinicopathological variables, and patient outcome.

The present study consists of two parts: (1) a comprehensive literature search to establish the frequency and clinical importance of mucin stains in SRC containing GC, and (2) a large cohort study in Asian and Caucasian GC where the histological phenotype was classified according to recently published consensus guidelines and the expression of several different mucin stains and its relationship to clinicopathological variables, patient outcome, and ethnicity was investigated.

## Materials and methods

### Literature review

**Fig. 1:**
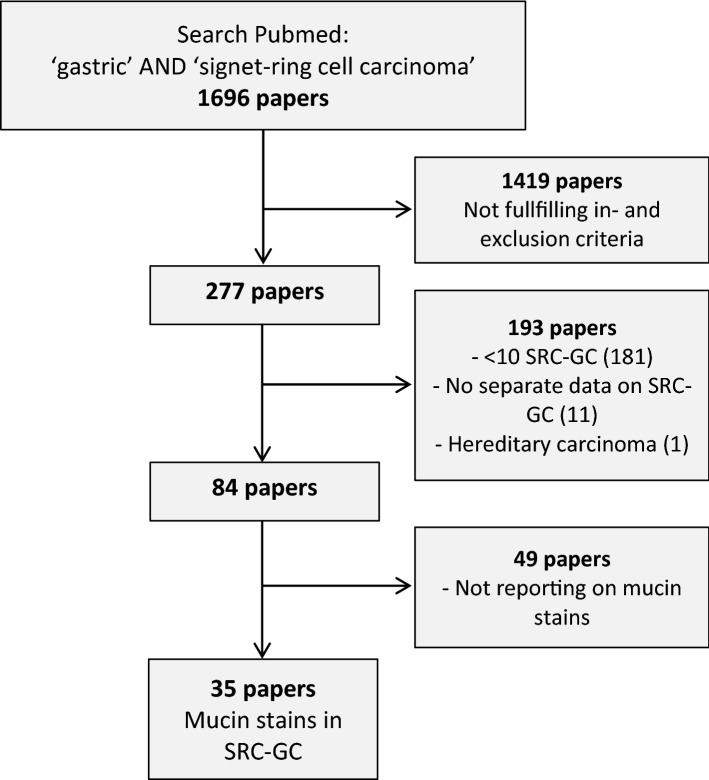
Flowchart of included papers in literature study. *SRC* signet-ring cell; *GC* gastric cancer

A comprehensive literature search was conducted in the PubMed database including all publications up to October 1st, 2018 using synonyms and MESH terms for ‘gastric’ and ‘signet-ring cell cancer’ (see Online Resource 1). The title and abstract of resulting articles were screened based on the following inclusion criteria: the abstract or title mentioned SRC-GC, and results from histochemical or immunohistochemical mucin stains were reported separately for SRC-GC (see Fig. 1). If a study provided both, data from an SRC-GC and non-SRC-GC cohorts, only the SRC-GC data were extracted and analysed. We excluded studies reporting results from less than 10 SRC-GC, referring to hereditary diffuse GC, metastases with SRCs or unknown primary tumour, cell culture-based studies, animal studies, diagnoses based on cytology, and letters to editors containing no additional information. We also excluded studies where we were unable to retrieve the full-text version of the article, articles written in languages other than English or Japanese, and articles reporting only on gastro-oesophageal junction adenocarcinomas.

From the included studies, we extracted information about the definition of SRC-GC, classification system used, frequencies of positivity for histochemical and immunohistochemical mucin stains, differences in mucin expression between SRC-GC and relationship between mucin expression in SRC-GC, patient outcome, and other clinicopathological factors.

### Gastric cancer cohort study

#### Patients

We included 851 Caucasian patients from Leeds Teaching Hospitals NHS Trust (LTHT), Leeds, UK, and 410 Asian patients from the Kanagawa Cancer Center Hospital (KCCH), Yokohama, Japan, diagnosed with adenocarcinoma of the stomach or gastro-oesophageal junction. All patients underwent potentially curative gastrectomy or gastro-oesophagectomy with lymph node dissection. 76 patients had neoadjuvant chemotherapy. Clinical variables (age, sex, treatment, overall and 5-year survival, and mortality status) and histopathological variables [(y)pT, (y)pN, grade of differentiation, and tumour location] were retrieved from hospital records and pathology reports. The study protocol was approved by the relevant local ethics committees.

Tissue microarrays (TMAs) had been previously constructed from the resection specimen [32]. Three 0.7 mm cores where sampled from each LTHT GC and two 1.2 mm cores from each KCCH GC. The cores were taken from areas with the highest tumour density in both cohorts. Four micron thick sections were cut and stained with haematoxylin–eosin (H&E) using a standard protocol and subjected to histochemical stains and immunohistochemistry as described below. Slides were scanned at × 40 magnification using an Aperio XT Scanner (Aperio Technologies, Vista, CA, USA) at the University of Leeds slide scanning facility and viewed using Aperio ImageScope (version 12.3.2.8013). Scoring of the immunohistochemical stains was performed independently by two observers (D.L. and K.K.), and discrepant scores were reviewed and discussed jointly until agreement was reached.

#### Histological classification

The H&E stain was used to classify GC based on 5th edition WHO Classification of Tumours of the Digestive System [31] in combination with the recently published consensus [25]. A poorly cohesive GC was defined as a cancer composed of isolated neoplastic cells or small aggregates of neoplastic cells [31]. To be classified as a mucinous GC, more than 50% of tumour area had to be occupied by pools of extracellular mucin [31]. GC not fulfilling the criteria for either poorly cohesive or mucinous GC were classified as non-poorly cohesive GC.

A classical SRC was defined as a cell with ample optically clear cytoplasmic mucin on H&E stain and eccentrically placed nucleus. To compare the mucin expression characteristics of SRCs in poorly cohesive GCs with lookalikes SRCs in non-poorly cohesive and mucinous GCs, we decided to quantify the proportion of SRCs in all histological subtypes based on the H&E appearance in 4 categories: < 10% SRCs, ≥ 10–50% SRCs, ≥ 50–90% SRCs, and ≥ 90% SRCs. This classification was performed blinded to (immuno)histochemical expression results or clinicopathological information.

#### Mucin histochemistry and immunohistochemistry

All GCs were stained for Alcian Blue (AB)-Periodic Acid-Schiff (PAS) combined with pan-cytokeratin (CK-ABPAS), MUC2, and MUC5AC. We chose ABPAS to differentiate between acidic and neutral mucin. ABPAS stain was combined with immunohistochemistry for pan-cytokeratin to distinguish between mucin within epithelial cells, mucin within non-epithelial cells such as macrophages and extracellular mucin. We chose MUC2 as intestinal-type mucin stain and MUC5AC as gastric-type mucin stain, since these stains were most frequently used in studies in the literature [33–40].

After deparaffinisation in xylene and rehydration following a standard protocol, the pan-cytokeratin stain was performed first (see below) followed by the ABPAS stain. We used 1% AB solution (pH 2.5), 1% periodic acid solution, and Schiff’s solution following our routine laboratory protocol. Slides were dehydrated, coverslipped, and scanned.

For all antibodies, antigen retrieval was performed in a microwavable pressure cooker using 10 mM citrate buffer, pH 6.0, and full pressure for 5 min. Hydrogen peroxide and egg white solution were used to block endogenous peroxidase activity and endogenous biotin, respectively. The sections were incubated with antibodies against MUC2 (dilution 1:100, clone CCP58; Agilent/Dako), MUC5AC (dilution 1:100, clone CLH2; Agilent/Dako), and pan-cytokeratin (dilution 1:200, clone AE1/AE3; Agilent/Dako) for 1 h at 37 °C. Dako Real streptavidin–biotin detection kit was used as detection system and 3,3′-Diaminobenzidine (DAB) as chromogen (Dako) according to the instructions of the manufacturer. Slides were counterstained with Mayer’s haematoxylin, dehydrated, coverslipped, and scanned.

#### Scoring of mucin stains

For all three stains (MUC2, MUC5AC, and ABPAS), a core with ≥ 10% stained tumour cells was classified as positive, irrespective of expression intensity. For this scoring, all tumour cells were considered irrespective of their morphology (signet-ring cell or not). If one of the cores of a case was classified as being positive, the whole case was classified as positive.

ABPAS-positive cores were initially subdivided into 5 categories: (1) AB positive; (2) PAS positive; (3) mixed positivity—same cells AB and PAS positive; (4) mixed positivity—different cells AB and PAS positive; (5) combination of categories 3 and 4. A case was classified as positive for both ABPAS, if AB and PAS positivity was seen in the same core (category 3–5) or if one core was AB positive (category 1) and another core was PAS positive (category 2). As the number of cases in certain ABPAS subcategories was very small, we subsequently combined groups (see Table 1).

**Table 1:** Descriptive statistics of the two cohorts

	Overall (*n* = 1261)		Caucasian (*n* = 851)		Asian (*n* = 410)		*p* value
	*n*	%	*n*	%	*n*	%	
Gender (male)	852	68	557	66	295	72	0.026*
Mean age (years)	66.6 (SD 11.0)		68.2 (SD 10.8)		63.3 (SD 10.7)		< 0.001*
T stage							< 0.001*
T1	131	11	96	12	35	9	
T2	146	12	84	11	62	15	
T3	307	26	252	33	55	13	
T4	600	51	343	44	257	63	
N stage							< 0.001*
N0	359	29	275	32	84	21	
N1	264	21	177	21	87	22	
N2	296	24	194	23	102	25	
N3	340	27	204	24	136	33	
Classification							0.641
PC < 10% SRCs	192	15	129	15	63	15	
PC > 10% SRCs	67	5	46	5	21	5	
Non-PC < 10% SRCs	905	72	608	71	297	72	
Non-PC > 10% SRCs	38	3	23	3	15	4	
MC < 10% SRCs	14	1	9	1	5	1	
MC > 10% SRCs	27	2	22	3	5	1	
Non-informative	18	1	14	2	4	1	
Mucins							
MUC2							0.043*
Negative	1050	83	698	82	352	86	
Positive	173	14	129	15	44	11	
MUC5AC							< 0.001*
Negative	844	67	544	64	300	73	
Positive	387	31	289	34	98	24	
ABPAS							0.037*
Negative	974	77	674	79	300	73	
Positive	255	20	159	19	96	23	
*AB positive	62	5	47	6	15	4	
*PAS positive	37	3	23	3	14	3	
*Mixed, same cells	7	0.6	2	0.2	5	1	
*Mixed, different cells	4	0.3	4	0.5	0	0	
*Mixed, both expressions	145	12	83	10	62	15	

*n* number of cases, *SRC* signet-ring cell, *PC* poorly cohesive cancer, *non-PC* non-poorly cohesive cancer, *MC* mucinous cancer, *SD* standard deviation

Furthermore, we created additional variables by combining the results of the different mucin stains (ABPAS, MUC2, and MUC5AC) (see Online Resource 2).

#### Statistical analyses

Data were analysed using SPSS Statistics for Windows version 25.0 (IBM, Armonk, NY, USA). Patient characteristics, histological tumour types, and mucin expression were compared between the LTHT and the KCCH cohorts using the Mann–Whitney *U* test for continuous variables and the Pearson Chi-square test for categorical variables. For the analysis of the association between histological phenotype and mucin expression, results from both cohorts (LTHT and KCCH) were combined. Associations between mucin expression and clinicopathological variables were assessed using the Kruskal–Wallis test. For survival analyses, individual mucin expression and combinations of mucin expressions (see Online Resource 2) were used. Kaplan–Meier survival analysis and log-rank test were used to compare 5-year and overall survival between patients with different histological tumour types or different mucin expression. 16 LTHT patients and 60 KCCH patients underwent neo-adjuvant chemotherapy, respectively. To determine whether neoadjuvant treatment influenced the results, all analyses were repeated excluding these 76 patients. As all associations remained the same, we report here results based on the whole cohort. *p* values < 0.05 were considered statistically significant.

## Results

### Literature review of mucin stains in gastric signet-ring cell cancers

The literature search in PubMed resulted in only 35 studies published over a period of 40 years (between 1977 and 2017) (see Fig. 1). The median number of SRC-GC patients per study was 37 (range 11–317), and the percentage of SRC-GC within individual studies ranged from 7 to 100%. The majority of studies (*n* = 25, 71%) originated from Asia.

#### Definition of gastric signet-ring cell cancers and classification system used

The definition of SRCs and the classification system used varied between studies (see Tables 2 and 3). Some authors provided a very detailed description mentioning intracellular accumulation of PAS, AB, and/or mucicarmine positive mucin [41–44] together with an eccentric nucleus as defining factors [44] or described ‘classical SRCs’ next to small SRCs with deep eosinophilic cytoplasm and a round larger hyperchromatic nucleus [45]. Other authors neither described the definition of SRC nor mentioned the classification system used. Some studies used cut-off values to define SRC-GC, either in concordance with the 2nd and 3rd editions WHO classification (50% rule) [35, 36, 38, 46–51] or using the 4th edition WHO classification [28] (predominant or exclusive rule without specific percentage) [41, 42]. One of the studies used a cut-off value of 90% to define a GC as SRC-GC [53].

**Table 2:** Results literature review reporting on individual immunohistochemical mucin stains

Author	Year	Number SRC-GC		Description SRC-GC	Classification tumour type used	Mucin stains (% positive^a^)				Relationship with survival	Comparison with other tumour types
		*n*	%			MUC2	MUC5AC	MUC6	EMA/MUC1		Yes/No
Sakamoto H	1997	38	17	No	No	66	NA	NA	7	Yes	Yes
Baldus SE	1998	62	48	No	WHO^b^, Lauren	27	NA	NA	60/45	Yes	Yes
Gürbüz Y	2002	31	19	Yes	No	81	100	35	52	NR	Yes
Akyürek N	2002	37	26	No	Borrmann, WHO^c^, Lauren	41	NA	NA	43	NS	Yes
Aihara R	2004	69	100	Yes	WHO^b^, JGCA	41	100	38	NA	Yes	No
Chu PG	2004	30	100	Yes	No	50	50	NA	17	NR	No
Zhang HK	2004	31	33	No	no	81	55	71	NA	NS	Yes
Nguyen MD	2006	21	100	Yes	No	48	38	29	24	NR	No
Sentani K	2008	21	100	Yes	No	71	48	NA	NA	NR	No
Chiaravalli AM	2009	24	20	Yes	Own	60	60	NA	NA	NR	Yes
Seki T	2009	35	100	Yes	Lauren, Nakamura	60	97	NA	NA	NR	No
Ilhan O	2010	19	7	No	WHO^c^, Lauren	95	11	NA	NA	NS	Yes
Bartley AN	2011	22	100	Yes	WHO^b^	57	100	NA	NA	NR	No
Yonezawa S	2012	69	35	No	JGCA	NA	NA	NA	6 to 97^e^	No	Yes
Konno-Shimizu M	2013	51	43	Yes	No	NA	High	NA	NA	NR	No
Terada T (II)	2013	30	100	Yes	WHO^d^	13	67	70	10 (MUC1)	NR	No
Terada T (III)	2013	30	100	Yes	WHO^d^	NA	NA	NA	57 (EMA)	NR	No
Nakajima T	2016	35	100	No	No	54	94	77	NA	No	No
Fujimoto A	2017	12	12	No	JGCA	50	75	NA	NA	NR	Yes
Kim YH	2017	317^f^	100	Yes	WHO^c^	36	50	44	NA	NR	No
Xiong ZF	2017	163	100	Yes	No	47	74	29	21	Yes	No

*n* number of cases, *%* percentage of all cases, *NA* not applicable, *NR* not reported, *NS* not specific for SRC, *SRC* signet-ring cell, *GC* gastric cancer, *WHO* World Health Organisation, *JGCA* Japanese Gastric Cancer Association

^a^Percentage rounded up

^b^2nd edition WHO

^c^3rd edition WHO

^d^4th edition WHO

^e^Used different antibodies

^f^92 instead of 317 cases were used for immunohistochemistry

**Table 3:** Results from studies in the literature reporting on mucin phenotypes based on immunohistochemical mucin stains

Author	Year	Number SRC-GC		Description SRC-GC	Classification tumour type used	Mucin phenotypes (% of total SRC-GC^a^)				Relationship with survival	Comparison with other tumour types
		*n*	%			*G*	*I*	GI/mixed	*U*		Yes/No
Bamba M	2001	54	100	Yes	JGCA	28	2	69	NA	Yes	No
Tsukashita S	2003	17	17	No	NR	77	0	0	23	NR	Yes
Aihara R	2004	69	100	Yes	WHO^b^, JGCA	60	0	41	0	Yes	No
Aihara R	2005	69	54	Yes	WHO^b^	47	2	46	5	Yes	Yes
Ohkura Y	2005	79	28	No	No	SRC: 81; mixed: 35	SRC: 2; mixed: 15	SRC: 16; mixed: 50	0	NR	No
Tian MM	2007	66	100	Yes	WHO^c^	26	15	47	12	Yes	No
Nakajima T	2016	35	100	No	No	0	0	35	0	NR	No
Xiong ZF	2017	163	100	Yes	No	39	18	42	NA	Yes	No

*n* number of cases, *%* percentage of all cases, *G* gastric phenotype, *I* intestinal phenotype, *GI* gastrointestinal phenotype, *U* unclassified, *NA* not applicable, *NR* not reported, *SRC* signet-ring cell, *GC* gastric cancer, *WHO* World Health Organisation, *JGCA* Japanese Gastric Cancer Association

^a^Percentages rounded up

^b^2nd edition WHO

^c^3rd edition WHO

#### Gastric signet-ring cell cancer and histochemical mucin stains

We identified 12 studies reporting on histochemical mucin stains in SRC-GC published between 1977 and 2013. The median number of included SRC-GC cases was 28 (range 11–102).

Different studies used single stain or combination of stains to classify SRC-GC (see Table 4).

**Table 4:** Histochemical mucin stains and detection purpose

Stains	Purpose/specific cell-type	References
Alcian blue (AB)	Identification of acid and neutral mucin; goblet cells	[39, 47–49, 51, 52, 55–57]
Periodic acid-Schiff (PAS) with or without diastase	Identification of acid and neutral mucin	[39, 47, 48, 50, 51, 56]
High iron diamine (HID) with AB	Differentiating sulphomucin from sialomucin	[51, 56]
Galactose oxidase-Schiff (GOS)	Identification of terminal β-galactose and β-*N*-acetylgalactosamine; gastric surface mucous cells	[56, 58]
Periodic acid-sodium borohydride-potassium hydroxide (PA-SB-PH)	Identification of sialic acid *O*-acylated side chain; goblet cells in large intestine	[56]
Mucicarmine	Acid mucin; goblet cells	[47, 48]
Paradoxical Concanavlin A stain (PCS)	Detection of stable class III mucin; cardiac glands, mucous neck cells, pyloric glands, and Brunner’s glands	[56, 58]
LNAase	Marker enzyme for small intestine and intestinal metaplasia	[55]

Three studies combined morphological features and histochemical mucin stains to classify SRC-GC into several subtypes. Kubota et al. [53] used AB, AB-PAS, and LNAase stains to classify 64 SRC-GC as type A (immature: PAS weak positive, AB negative, LNAase positive, small cell size, and high nuclear/cytoplasmic ratio), type B (intermediate: stronger PAS positivity, AB negative or weak positive, LNAase positive, and smaller nuclear/cytoplasmic ratio) or type C (mature: PAS strong positive, AB positive, and eccentric nucleus). The C-type was further divided in C1 and C2 subtypes with the C1 subtype showing weaker AB positivity combined with LNAase positivity and the C2 subtype showing stronger AB positivity combined with negative LNAase expression. Akamatsu et al. [54] used five histochemical stains (AB-PAS, HID-AB, GOS, PA-SB-PH-PAS, and PCS) to classify 31 SRC-GC into 6 subtypes (1) surface mucous cell-type; (2) mucous neck cell-pyloric cell-type; (3) goblet cell (small intestine)-type; (4) goblet cell (large intestine)-type; (5) microcyst-type and (6) unclassified. Although different SRC sub types could be present in the same tumour, types 1, 2, 3, and 4 were observed most frequently as the dominant cell type. In addition, the authors described the presence of the so-called intramucosal laminated structures (ILS) in SRC cancer distinguishing three types: complete (upper, middle, and lower layers of the mucosa), incomplete (upper and middle layers), and inverted (middle and lower layer) based on morphology and histochemical mucin expression. Tatematsu [55, 56] combined histochemical stains for PCS, GOS, and sialidase GOS with immunohistochemical stains for pepsinogen I and II to classify 127 SRC-GC as gastric phenotype, intestinal phenotype, or mixed gastrointestinal phenotype. The gastric phenotype was the most prevalent subtype (in both studies approximately 74%).

However, the above mentioned detailed histomorphological subclassifications of SRC-GC have not been validated in any subsequent studies and the relationship with clinical variables has only been investigated by Akamatsu [54] who did not find any associations.

#### Frequency of histochemical mucin positivity in gastric signet-ring cell cancers

Fujiyoshi et al. [43] categorised SRCs into ‘classical’ (33.3%) and ‘non-classical’ based on AB positivity, whereas Bakkelund et al. [44] used PAS positivity as defining feature of SRCs, although the amount of PAS positive material varied between SRCs in the same GC and between GCs. Work by Terada et al. [41, 42] suggested that SRCs were positive for PAS-D, AB and mucicarmine, whereas Santini et al. [45] showed that 51% of SRC-GCs were positive for PAS, AB, and high-iron diamine (HID), 12% positive for AB or PAS and HID, and 37% showed PAS positivity only. Takenoshita et al. [57] compared PAS and AB positivity between ‘pure’ SRC-GC and tubular or poorly differentiated GC containing SRCs. All pure SRC-GC were strongly PAS positive and 71% were also strongly AB positive. Interestingly, the AB and PAS positivity rate was similar in SRC containing tubular or poorly differentiated GCs, and only the expression intensity was found to be lower. Furthermore, SRC-GC seemed to have stronger AB expression compared to PAS expression (*p* < 0.01) [57].

In summary, the frequency of AB positive SRC-GC varied from 56% [45] to 64% [58], and PAS positivity varied from 61% [57] to 95% [45]. One study described HID positivity in 63% of GCs [45].

#### Relationship of histochemical mucin positivity with clinicopathological variables

We did not find any study investigating the relationship between AB and/or PAS positivity and clinicopathological variables or patient outcome.

A single study combined histochemical mucin stains (PCS, GOS, and sialidase GOS) and immunohistochemical stains [pepsinogen II, SH-9 (surface mucous cell stain), and TKH-2 (goblet cells and parietal cell stain)] to define the phenotype of 203 SRC-GC as gastric [> 90% surface mucous cell type or pyloric gland cell type, *n* = 130 (64%)], intestinal [> 90% goblet cell type or microcyst type, *n* = 4 (2%)], or mixed gastrointestinal [10–90% gastric and/or intestinal cell types, *n* = 69 (34%)] [46]. The proportion of gastric phenotype SRC-GC decreased with increasing depth of invasion, whereas the proportion of mixed phenotype SRC-GC increased with depth of invasion. This could suggest that the progression of SRC-GC may be associated with a phenotypic shift from gastric to intestinal-type mucin expression.

#### Immunohistochemical stains for mucin expression of gastric signet-ring cell cancers

Twenty-six studies using immunohistochemistry were published between 1997 and 2017, and the median (range) number of SRC-GC was 31 (12–317). The results are summarised in Tables 2 and 3.

We noted a wide variation of cut-off values used to classify a cancer as being positive for a particular marker which most likely explains the wide range in reported positivity frequencies. MUC4, STn, and trefoil factor family peptide (TFF) 1 and TFF3 were only investigated in a single study and reported to be positive in 57% [58], 57% to 71% [59], 33.3% and 100% [60] SRC-GC, respectively.

The frequency of single mucin stain positive SRC-GC ranged from 13% [41] to 95% [51] for MUC2, 11% [51] to 100% [35, 49, 61] for MUC5AC, 29% [58] to 71% [62] for MUC6 and 6% [63] to 97% [63] for EMA/MUC1. A single study by Kim et al. [50] compared the frequency of mucin expression between pure SRC-GC and SRC-GC combined with < 50% tubular or papillary component and did not find any differences for MUC2, MUC5AC, or MUC6.

Some studies suggested a relationship between mucin expression and depth of invasion. MUC5AC expression was seen in tumour cells in the superficial [33, 35, 49] part of the gastric wall, whereas tumour cells positive for MUC6 [35] or PCS III [33] were more frequently found in deeper parts of the wall. The location of MUC2 positive tumour cells appeared to be more variable and could be superficially [49], central/marginal [35], or in a mosaic pattern [33].

Several studies [33–40] investigated combinations of mucin stains in SRC-GC (see Table 3) and described four SRC-GC phenotypes: gastric (G), intestinal (I), gastrointestinal/mixed (GI), and unclassified (UC). The G-type showed expression of one or more gastric mucin stains (MUC5AC and/or MUC6 and/or PCSIII and/or M-GGMC-1) and absence of intestinal-type mucin stain MUC2; the I-type showed expression of intestinal mucin stains and absence of gastric mucin stains; the GI-type showed a combination of stains and the unclassified type is negative for any mucin stains (for details, see Online Resource 3). However, the reported frequency of these phenotypes in SRC-GC varied: G-type ranged from 0% [39] to 81% [37], I-type from 0% [34, 35, 39] to 18% [40], and GI-type 0% [34] to 69% [33]. Aihara et al. [36] found a difference in mucin phenotype when comparing SRC-GC with non-SRC-GC (SRC-GC: 41 G-type, 28 GI-type; non-SRC-GC: 20 G-type, 31 GI-type, *p* = 0.029).

Seki et al. [64] divided 35 intramucosal SRC-GC in three groups based on the type of ILS (as described by Akamatsu et al. [54]): complete type only (group A—40%), both complete and incomplete type (group B—49%), and incomplete type only (group C—11%). MUC2 expression was different (29%, 76%, and 100% of GC in groups A, B, and C, respectively. *p* = 0.0006).

#### Relationship between immunohistochemical mucin stains and clinicopathological variables including patient outcome

Some studies reported a poorer outcome for patients with MUC1 positive SRC-GC [48, 60]. GI and I-type SRC-GC were associated with poorer overall outcome compared to G and UC-type (31.82% vs. 68.75%, *p* = 0.0146) [38]. No association was found between patient outcome and MUC2 or STn positivity [60].

MUC2 positive or GI-type SRC-GC was associated with larger tumour diameter [35, 36, 38], increased depth of invasion [35, 36, 38, 40], presence of lymph-node metastases [38], or lymphovascular invasion [36, 38, 40]. Patients with G-type SRC-GC had smaller tumours, lower rates of lymph-node metastasis, or vascular invasion compared to other phenotypes (both *p* < 0.01) [38]. Furthermore, Xiong et al. [40] found that MUC5AC expression was inversely associated with depth of invasion. Bamba et al. [33] showed that mucosal tumour size was related to abundance of I-type tumour cells, whereas no such relationship was seen for tumour cells in deeper parts of the wall.

To the best of our knowledge, the relationship between mucin positivity and response to therapy has not been investigated.

#### Comparison of immunohistochemical mucin stains between gastric signet-ring cell cancer and other histological tumour types

The relationship between immunohistochemical mucin expression and histological tumour type in GC is still controversial. Some studies reported that tubular/papillary/glandular adenocarcinomas were more frequently MUC1 [47, 48, 59, 61, 62], MUC2 [47, 48] and/or MUC5AC [51] positive compared to SRC-GC. Whereas other studies found that SRC-GC were more often MUC2 [51, 59, 60, 65], TFF3 [60], or MUC5AC [61, 65] positive compared to other types of GC, Ilhan et al. [51] found no relationship of MUC1 expression and histological tumour type. Zhang [62] did not find an association between MUC2, MUC5AC, and MUC6 expression and histological tumour type, whereas other studies showed that MUC2 expression was associated with mucinous cancers [51, 61, 62].

In conclusion, the existing number of studies on (immuno)histochemical mucin expression in SRC-GC is limited, and results are controversial most likely related to sample size, the use of different mucin stains, or combinations of stains and variable cut-offs. We noted differences in frequencies of mucin positivity in SRC-GCs as well as differences in the reported association with clinicopathological variables including patient outcome. The majority of published studies were performed using material from Asian GC patients; thus, it is not clear whether results in Caucasian patients would be similar.

All the above motivated us to complement the comprehensive literature review with a large cohort study on more than 1000 patients with gastric or gastro-oesophageal cancers.

### Results from our own gastric and gastro-oesophageal cancer cohort study

This study included material from 958 (76%) patients with GC and 303 (24%) patients with junctional/lower oesophageal adenocarcinoma. For clinicopathological and demographic cohort characteristics, see Table 1. This cohort included 851 Caucasian patients (LTHT cohort: 709 gastric and 142 junctional/lower oesophageal cancer) and 410 Asian patients (KCCH cohort: 249 gastric and 161 junctional/lower oesophageal cancer).

#### Frequency of mucin positivity

Using the presence of expression in more than 10% tumour cells as cut-off for all 3 stains (MUC2, MUC5AC, AB/PAS), 670 (53%) cancers were classified as ‘triple negative’. 172 (14%) cancers were classified as MUC2 positive, 383 (31%) MUC5AC positive, and 253 (20%) AB and/or PAS positive (see Table 1). 145 AB and/or PAS positive cancers showed a mixed expression with both, the same and different cells being positive for AB and/or PAS. 330 (26%) cancers were positive for only one of the three mucin stains (48 (4%) only MUC2 positive, 217 (17%) only MUC5AC positive, and 65 (5%) only ABPAS positive). 176 (14%) cancers were positive for two mucin stains whereby combined positivity of MUC5AC and ABPAS was most frequently seen (94 (8%) cancers). 42 (3%) cancers were triple positive (see also Online Resource 2).

#### Histological phenotypes and associations with mucin stains

**Fig. 2:**
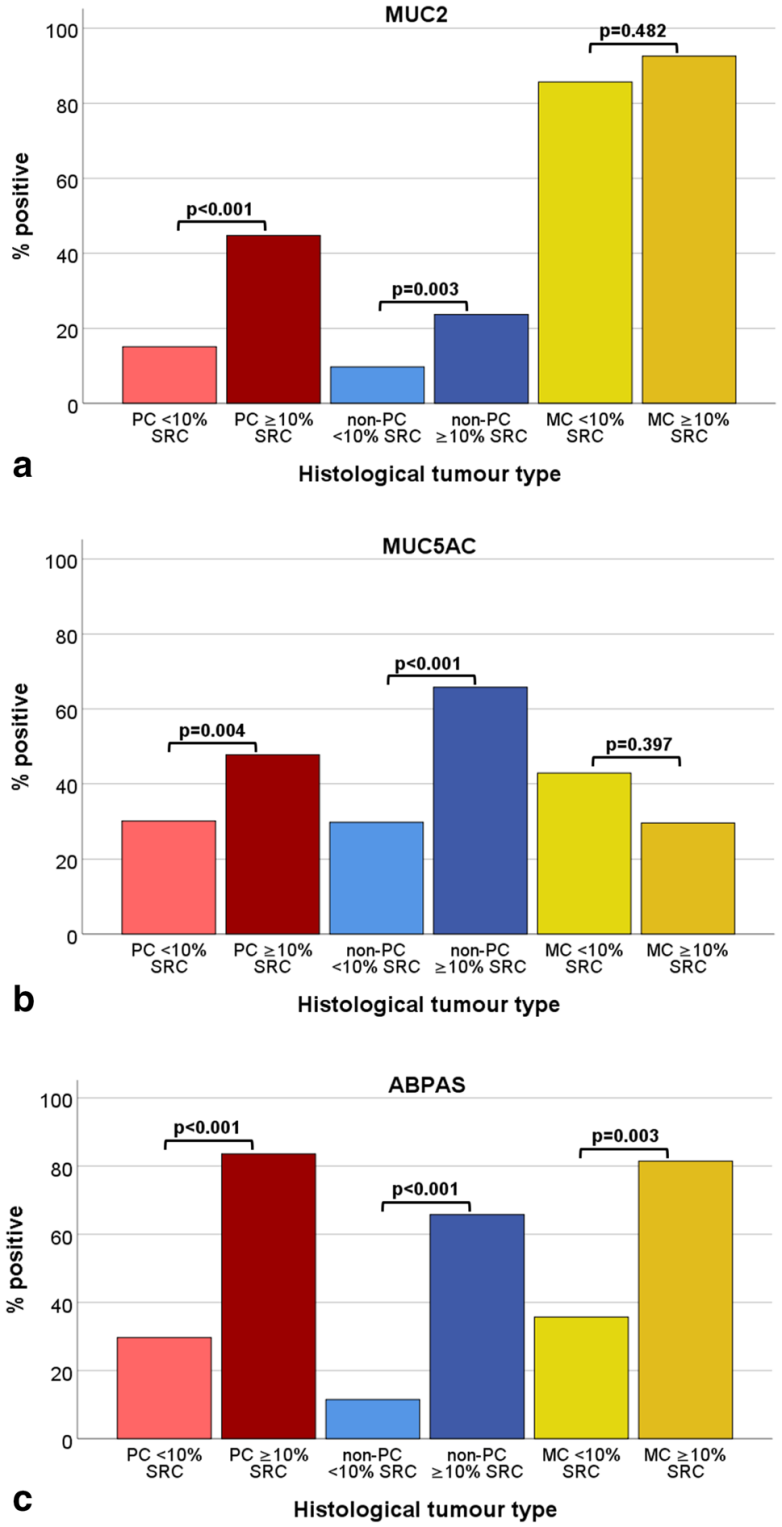
Mucin expression in association with histological tumour type. **a** MUC2; **b** MUC5AC; **c** ABPAS. For PC and non-PC cases with ≥ 10%, RCs showed more MUC2 (*p* < 0.001 and *p* = 0.003), MUC5AC (*p* = 0.004 and *p* < 0.001), and ABPAS (*p* < 0.001 and *p* < 0.001) positivity compared to cases with < 10% SRCs. MC showed high MUC2 expression. MC with ≥ 10% SRCs were more often ABPAS positive compared to MC with < 10% SRCs (*p* = 0.003). *PC* poorly cohesive cancer; *non-PC* non-poorly cohesive cancer; *MC* mucinous cancer; *SRC* signet-ring cell

259 (21%) cancers were classified as poorly cohesive, 943 (75%) as non-poorly cohesive, and 41 (3%) as mucinous. An overview of results from the non-poorly cohesive and mucinous cancers can be found in Table 1 and Fig. 2. In the group of poorly cohesive cancers, 192 (74%), 36 (14%), 21 (8%), and 10 (4%) cancers contained < 10% SRCs, ≥ 10–50% SRCs, ≥ 50–90% SRCs, and ≥ 90% SRCs, respectively. Signet-ring cells were seen in mucinous cancers: < 10% SRCs (*n* = 14, 34%), ≥ 10–50% SRCs (*n* = 12, 29%), ≥ 50–90% SRCs (*n* = 9, 22%), and ≥ 90% SRCs (*n* = 6, 15%). As expected, the number of non-poorly cohesive cancers with cells looking like signet-ring cells was very low, only 38 (3.8%) cancers contained more than 10% lookalike SRCs, and none of the non-poorly cohesive cancers contained more than 50% lookalike SRCs.

Because of relatively small number of cases in subgroups, we compared poorly cohesive cancer with < 10% SRCs (*n* = 192, 15%), poorly cohesive cancer with ≥ 10% SRCs (*n* = 67, 5%), non-poorly cohesive cancer with < 10% SRCs (*n* = 905, 72%), non-poorly cohesive cancer with ≥ 10% SRCs (*n* = 38, 3%), mucinous cancer with < 10% SRCs (*n* = 14, 1%), and mucinous cancer with ≥ 10% SRCs (*n* = 27, 2%) (see also Table 1).

Both, poorly cohesive and non-poorly cohesive cancers with ≥ 10% SRCs, were more frequently MUC2, MUC5AC, or ABPAS positive compared to cancers with < 10% SRCs (see Fig. 2). MUC2 was highly expressed in all mucinous cancers irrespective of the percentage of SRCs. Mucinous cancers with < 10% SRCs were less often ABPAS positive compared to mucinous cancers with ≥ 10% SRCs.

#### Comparison between the LTHT cohort and the KCCH cohort

The proportion of cancers with a particular histomorphological phenotype was similar in the LTHT and KCCH cohorts (see Online Resource 4). In both cohorts, non-poorly cohesive cancers were the most common histological subtype [LTHT *n* = 608 (71%); KCCH *n* = 297 (72%)]. MUC2- and MUC5AC-positive cancers were more frequent in the LTHT cohort (MUC2 LTHT *n* = 129 (15%) vs. KCCH *n* = 44 (11%), *p* = 0.043; MUC5AC LTHT *n* = 289 (34%) vs. KCCH *n* = 98 (24%), *p* < 0.001), whereas ABPAS-positive cancers were more frequent in the KCCH cohort (KCCH *n* = 96 (23%) vs. LTHT *n* = 159 (19%); *p* = 0.037) (see also Table 1).

**Fig. 3:**
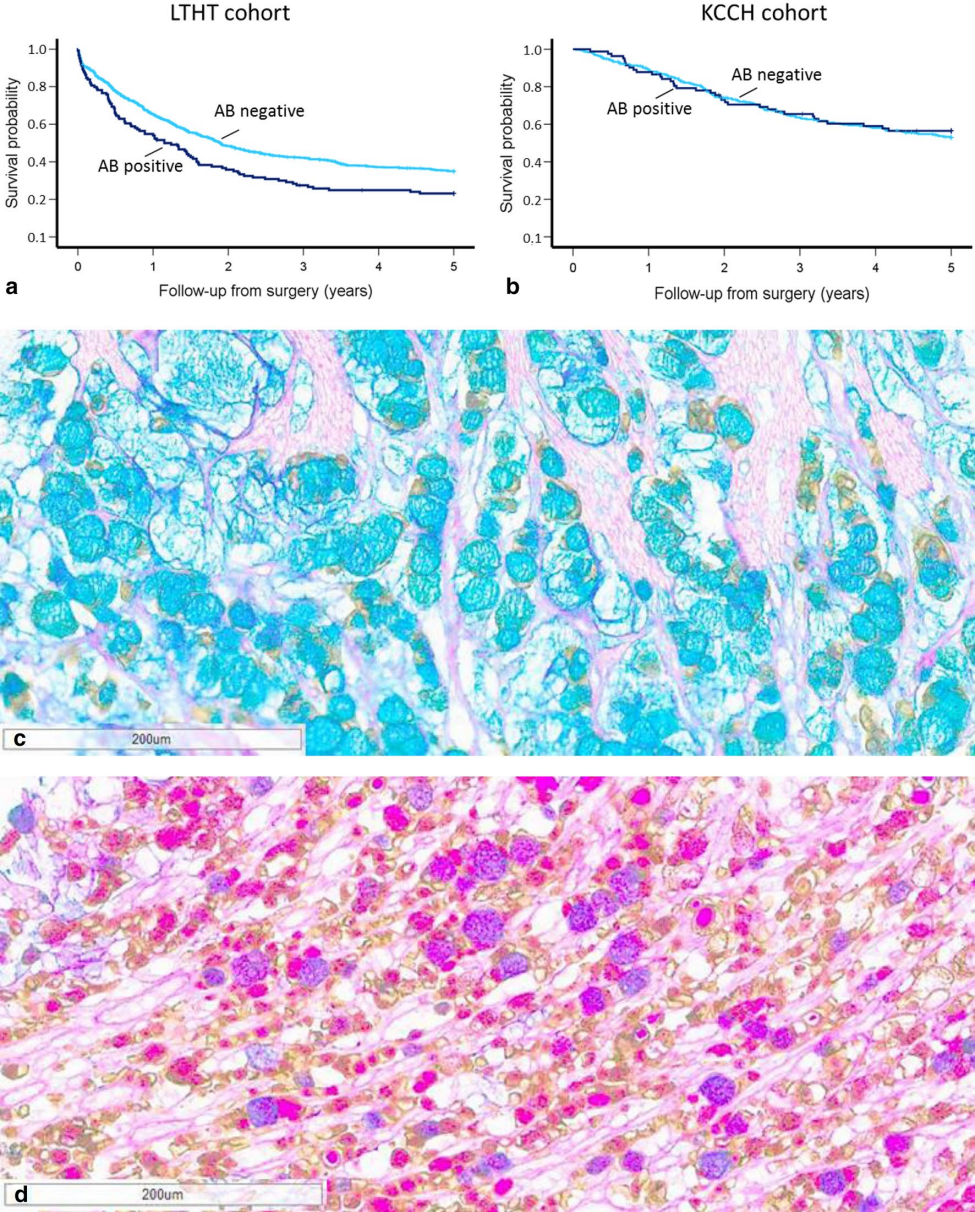
Kaplan–Meier plots showing probability of 5-year survival stratified by AB expression and cohort **a** and **b** Kaplan–Meier survival analysis showed in the LTHT cohort significantly worse outcome in AB positive GC (*p* = 0.002), this was not seen in the KCCH cohort. **c** Example of CK-ABPAS stain showing positivity for AB (blue staining). **d** Example of CK-ABPAS stain showing positivity for PAS and combined AB-PAS positivity (pink and purple staining respectively)

AB positivity was associated with worse 5-year survival only in the LTHT cohort (*p* = 0.002) (see Fig. 3). PAS, MUC2, or MUC5AC positivity was not related to outcome in any of the cohorts. Presence of combined ABPAS positivity, MUC2 positivity, and MUC5AC negativity (*n* = 35, 4%) was related to poorer 5-year survival in the LTHT cohort (*p* = 0.002) (see Fig. 3 and Online Resource 5). No other associations with patient outcome were found in either cohort.

## Discussion

There is an on-going debate whether patients with signet-ring cell (SRC) type gastric or gastro-oesophageal cancer have a different prognosis and response to chemotherapy [12–18] and, therefore, should be treated differently to patients with other gastric cancer (GC) subtypes. The inconsistent results reported in the literature could be related to the fact that the histopathological classification of SRC-GC can be challenging. This is due to the variable morphological appearances of individual SRCs [54–56] which may or may not be recognised as SRCs by some investigators [54, 54], as well as changing criteria in the WHO classification over the last decades [27–31]. Whilst an expert panel recently proposed cut-offs to enable separating pure SRC-GC (≥ 90% SRCs) from GC with an SRC component, a biomarker to unequivocal identify SRC-GC would be of potential great value to clinicians and patients.

In search of a potential promising biomarker, we decided to focus on mucin-related stains as mucin appeared to be a defining characteristic of SRCs. Our group was the first to conduct a comprehensive literature review into the reported frequency and clinical importance of different mucin stains in SRC-GC. To our surprise, the number of studies describing (immuno)histochemical mucin expression in SRC-GC was very limited and results varied between studies making firm conclusions difficult. However, the existing literature seemed to suggest that mucin is not only present in SRC-GC, but can also be seen in other types of GC [47, 51, 59, 61, 62]. Furthermore, some SRC-GC appeared to contain no mucin [34, 35, 39]. Similarly to the well described morphological heterogeneity of GC, we found evidence that combinations of different kinds of mucins can be seen in the same GC [33, 35–40, 45]. Our literature review supports the previous suggestion of an expert panel that inconsistent clinicopathological findings can at least partly be explained by differences in the histological haematoxylin–eosin-based classification of SRC-GC together with different cut-off values used for considering a stain positive. Furthermore, even if the same cut-off value was used, results remained contradictory [39, 40, 48, 49, 52, 62, 63] which could be related to the use of different primary antibodies [64]. When comparing reported results from Asian and Caucasian SRC-GC [34, 39, 41, 49, 58, 62], we saw similar wide ranges of mucin positivity, suggesting that differences might not be simply related to ethnic origin of the cancers. Sampling of the tumours (luminal versus centre versus invasive front) could potentially explain different results in mucin expression as it has been reported by several investigators that intramucosal SRC-GC showed a ‘layered structure’ both morphologically and (immuno) histochemically [33, 35, 49]. Mucin characteristics of SRC-GC varied depending on tumour size and disease stage [46, 66], thus results might vary depending on the case selection for the study [35, 36, 38, 46].

In order to close the gap in the current literature revealed by our comprehensive review, we decided to conduct our own gastric and gastro-oesophageal cohort study into mucin stains. Our study is the largest study to date where all cancers were re-classified in a standardised manner according to WHO classification and recent consensus [25]. Furthermore, our study is the first study to include both, Asian and Caucasian patients, enabling us to directly compare patient characteristics, histological tumour types, mucin expression, and relationship between mucin expression and patient outcome. Previous studies had either investigated Caucasian cohorts or Asian cohorts.

Our study provides further evidence that there is no (immuno)histochemical mucin stain unique to SRC cancer, since (1) a proportion of SRC containing GC was negative for the (immuno)histochemical mucin stains and (2) a relatively large percentage of GC without SRCs were positive for one or more mucin stains, similarly to what had been reported in the literature [47, 48, 51, 59–62, 65]. However, this is the first study to suggest that the mucin expression might be related to the quantity of SRCs within a given tumour as we saw more frequently mucin expression in poorly cohesive GC containing ≥ 10% SRCs.

When directly comparing Caucasian (LTHT) and Asian (KCCH) GC cohorts, MUC2 and MUC5AC positivity was more frequent in the LTHT cases, whereas ABPAS positivity was more frequent in the KCCH cases. This is the first study to report a relationship between AB positivity and outcome in GC patients. Most interestingly, AB positivity alone or in combination with other mucin expressions was only related to poor outcome in Caucasian GC providing further support for the hypothesis that different outcome in Caucasian and Asian GC patients may be related to an underlying biological difference [67]. As AB stains acidic mucins which are considered to be present in an intestinal phenotype, our finding would support the described association between (gastro) intestinal mucin phenotype and unfavourable outcome [38]. Due to relatively low patient numbers in mucin-defined subgroups, we were unable to explore whether the AB expression related outcome difference between cohorts was related to a different disease stage mix.

Based on the literature review and the results of our cohort study, we had to reject our first working hypothesis and conclude that SRC containing GC do not have a different mucin expression compared to non-SRC-GC. Further studies are needed to address this clinical need. On the other hand, results reported in the literature and from our own cohort study confirmed a relationship between SRC mucin expression and patient outcome in the surgery alone setting. Furthermore, our cohort study suggests that irrespective of histological phenotype, the mucin expression is different in Caucasian and Asian GC patient and is associated differently with outcome in different ethnic groups.

We recommend further studies comparing Caucasian with Asian GC to validate our findings and explore underlying molecular mechanisms for difference in mucin expression and outcome. Also, we recommend investigating whether the different mucin expression in SRC containing GC is related to variable treatment response.

## Electronic supplementary material

Below is the link to the electronic supplementary material.

Supplementary file1 (DOCX 16 kb)

Supplementary file2 (DOCX 20 kb)

Supplementary file3 (DOCX 21 kb)

Supplementary file4 (DOCX 21 kb)

Supplementary file5 (DOCX 100 kb)
