# Supplementary material for: Mucin expression in gastric- and gastro-oesophageal signet-ring cell cancer: results from a comprehensive literature review and a large cohort study of Caucasian and Asian gastric cancer
Source: Gastric Cancer. 2020 Jun 2;23(5):765–79. doi: 10.1007/s10120-020-01086-0 (PMC7438382; doi:10.1007/s10120-020-01086-0)
Supplement: Supplementary file 1 — Supplementary file1 (DOCX 16 kb) [file 10120_2020_1086_MOESM1_ESM.docx]

**Online Resource 1: Search terms used in PubMed**

| **Step** |  |
| --- | --- |
| **1** | ((Stomach[MeSH Terms]) OR Stomach[Title/Abstract]) OR Gastric[Title/Abstract] Filters: English; Field: Title/Abstract |
| **2** | ((((((Neoplasm[MeSH Terms]) OR Neoplasm[Title/Abstract]) OR Carcinoma[MeSH Terms]) OR Carcinoma[Title/Abstract]) OR Adenocarcinoma[MeSH Terms]) OR Adenocarcinoma[Title/Abstract]) OR Cancer[Title/Abstract] Filters: English; Field: Title/Abstract |
| **3** | ((((Carcinoma, Signet Ring Cell[Title/Abstract]) OR Carcinoma, Signet Ring Cell[MeSH Terms]) OR Signet Ring[Title/Abstract]) OR Signet Ring Cell[Title/Abstract] Filters: English; Field: Title/Abstract |
| **4** | Combination 1 AND 2 AND 3 |
