# Supplementary material for: Mucin expression in gastric- and gastro-oesophageal signet-ring cell cancer: results from a comprehensive literature review and a large cohort study of Caucasian and Asian gastric cancer
Source: Gastric Cancer. 2020 Jun 2;23(5):765–79. doi: 10.1007/s10120-020-01086-0 (PMC7438382; doi:10.1007/s10120-020-01086-0)
Supplement: Supplementary file 2 — Supplementary file2 (DOCX 20 kb) [file 10120_2020_1086_MOESM2_ESM.docx]

**Online Resource 2:**

**Number of cases in the subgroups based on combinations of mucin stains**

|  | **Overall (n=1261)** | | **Caucasian (n=851)** | | **Asian (n=410)** | |
| --- | --- | --- | --- | --- | --- | --- |
| **Mucin expression** | **n** | **%** | **n** | **%** | **n** | **%** |
| **Triple positive** | 42 | 3 | 34 | 4 | 8 | 2 |
| **ABPAS+ MUC2+ MUC5AC-** | 52 | 4 | 35 | 4 | 17 | 4 |
| **ABPAS+ MUC2-MUC5AC+** | 94 | 8 | 63 | 8 | 31 | 8 |
| **ABPAS+ MUC2-MUC5AC-** | 65 | 5 | 26 | 3 | 39 | 10 |
| **Triple negative** | 670 | 53 | 442 | 53 | 228 | 58 |
| **ABPAS- MUC2+ MUC5AC-** | 48 | 4 | 37 | 5 | 11 | 3 |
| **ABPAS- MUC2-MUC5AC+** | 217 | 17 | 167 | 20 | 50 | 13 |
| **ABPAS- MUC2+ MUC5AC+** | 30 | 2 | 23 | 3 | 7 | 2 |

n = number of cases
