# Supplementary material for: Mucin expression in gastric- and gastro-oesophageal signet-ring cell cancer: results from a comprehensive literature review and a large cohort study of Caucasian and Asian gastric cancer
Source: Gastric Cancer. 2020 Jun 2;23(5):765–79. doi: 10.1007/s10120-020-01086-0 (PMC7438382; doi:10.1007/s10120-020-01086-0)
Supplement: Supplementary file 3 — Supplementary file3 (DOCX 21 kb) [file 10120_2020_1086_MOESM3_ESM.docx]

**Online Resource 3:**

Details of studies of the iterature review reporting on mucin phenotypes based on immunohistochemical mucin stains – definition for phenotypes

|  |  | **Mucin phenotypes** | | | |
| --- | --- | --- | --- | --- | --- |
| **Author** | **Year** | **Gastric (G)** | **Intestinal (I)** | **Gastrointestinal (GI)/mixed** | **Unclassified (U)** |
| **Bamba M** | 2001 | MUC5AC and/or PCSIII, no MUC2 | MUC2, no MUC5AC or PCSIII | Other than G or I-type | not applicable |
| **Tsukashita S** | 2003 | MUC5AC and/or MUC6, no MUC2 or CD10 | MUC2 and/or CD10, no MUC5AC or MUC6 | Both G and I type markers | No markers |
| **Aihara R** | 2004,  2005 | MUC5AC and/or MUC6, no MUC2 | MUC2, no MUC5AC or MUC6 | Both G and I type markers | No markers |
| **Ohkura Y** | 2005 | MUC5AC and/or M-GGMC-1, no MUC2 | MUC2, MUC5AC and/or M-GGMC-1 | Both G and I type markers | No markers |
| **Tian MM** | 2007 | MUC5AC and/or MUC6, no MUC2, villin, CDX2 or Li-cadherin | MUC2 and/or villin and/or CDX2 and/or Li-cadherin, no MUC5AC or MUC6 | Both G and I type markers | No markers |
| **Nakajima T** | 2016 | MUC5AC or MUC6, no MUC2 or CD10 | MUC2 or CD10, no MUC5AC or MUC6 | Both G and I type markers | No markers |
| **Xiong ZF** | 2017 | MUC1 and/or MUC5AC and/or MUC6, no MUC2 or CDX2 | MUC2 and/or CDX2, no MUC1, MUC5AC or MUC6 | Both G and I type markers | not applicable |
