# Supplementary material for: Mucin expression in gastric- and gastro-oesophageal signet-ring cell cancer: results from a comprehensive literature review and a large cohort study of Caucasian and Asian gastric cancer
Source: Gastric Cancer. 2020 Jun 2;23(5):765–79. doi: 10.1007/s10120-020-01086-0 (PMC7438382; doi:10.1007/s10120-020-01086-0)
Supplement: Supplementary file 4 — Supplementary file4 (DOCX 21 kb) [file 10120_2020_1086_MOESM4_ESM.docx]

**Online Resource 4:**

Number of cases in subgroups based on histological tumour type

|  | **Overall (n=1261)** | | **Caucasian (n=851)** | | **Asian (n=410)** | |
| --- | --- | --- | --- | --- | --- | --- |
| **Classification** | **n** | **%** | **n** | **%** | **n** | **%** |
| PC <10% SRCs | 192 | 15 | 129 | 15 | 63 | 15 |
| PC ≥10-50% SRCs | 36 | 3 | 25 | 3 | 11 | 3 |
| PC ≥50-90% SRCs | 21 | 2 | 14 | 2 | 7 | 2 |
| PC ≥90% SRCs | 10 | 0.8 | 7 | 0.8 | 3 | 0.7 |
| non-PC <10% SRCs | 905 | 72 | 608 | 71 | 297 | 72 |
| non-PC ≥10-50% SRCs | 30 | 2 | 18 | 2 | 12 | 3 |
| non-PC ≥50-90% SRCs | 8 | 0.6 | 5 | 0.6 | 3 | 0.7 |
| non-PC ≥90% SRCs | 0 | 0 | 0 | 0 | 0 | 0 |
| MC <10% SRCs | 14 | 1.1 | 9 | 1 | 5 | 1 |
| MC ≥10-50% SRCs | 12 | 1.0 | 10 | 1 | 2 | 0.5 |
| MC ≥50-90% SRCs | 9 | 0.7 | 8 | 0.9 | 1 | 0.2 |
| MC ≥90% SRCs | 6 | 0.5 | 4 | 0.5 | 2 | 0.5 |
| Non-informative | 18 | 1.4 | 14 | 2 | 4 | 1 |

n = number of cases; SRC = signet-ring cell; PC = poorly cohesive cancer; non-PC = non-poorly cohesive cancer;

MC = mucinous cancer
