# Supplementary material for: Mucin expression in gastric- and gastro-oesophageal signet-ring cell cancer: results from a comprehensive literature review and a large cohort study of Caucasian and Asian gastric cancer
Source: Gastric Cancer. 2020 Jun 2;23(5):765–79. doi: 10.1007/s10120-020-01086-0 (PMC7438382; doi:10.1007/s10120-020-01086-0)
Supplement: Supplementary file 5 — Supplementary file5 (DOCX 100 kb) [file 10120_2020_1086_MOESM5_ESM.docx]

**Online Resource 5:** Kaplan-Meier plots showing 5-year survival stratified by mucin expression and cohort


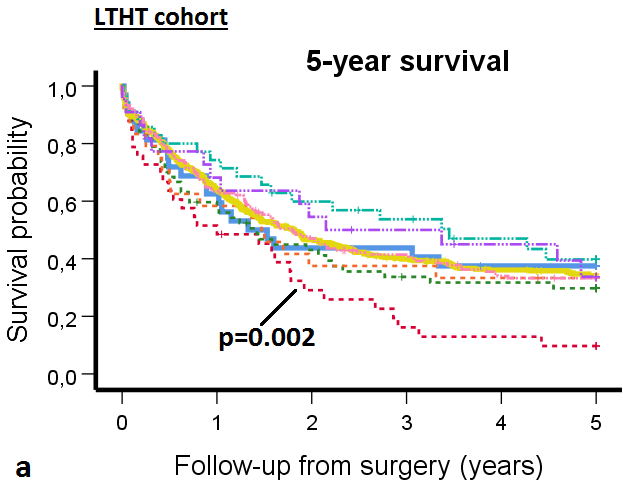


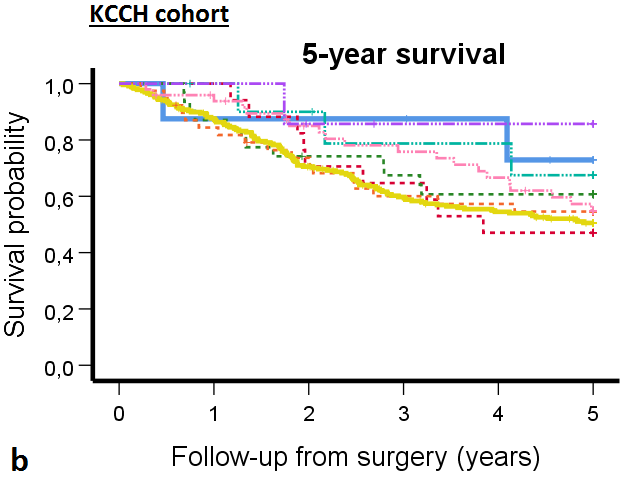


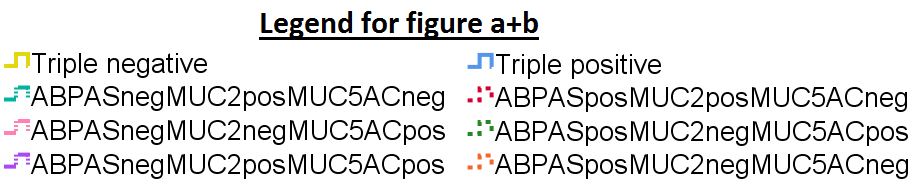


a + b. Kaplan-Meier survival analysis showed in the LTHT cohort a significantly worse 5-year survival in combined ABPAS positive, MUC2 positive, MUC5AC negative GC compared to the rest of the cases (p=0.002). No significant association was seen in the KCCH cohort (p=0.739).
